# *Mycobacterium fortuitum*: A Neglected Cause of Culture-Negative Prosthetic Valve Endocarditis and a Literature Review

**DOI:** 10.3390/idr18020023

**Published:** 2026-03-13

**Authors:** Selen Şahin, İrem Tümkaya Kılınç, Eda Yüksel, Çağla Mehmet, Bedia Dinç, Emine Alp Meşe

**Affiliations:** 1 Department of Infectious Diseases and Clinical Microbiology, Ankara Bilkent City Hospital, 06800 Ankara, Türkiye; selen_sahin06@hotmail.com (S.Ş.); edayuksel2097@gmail.com (E.Y.); caglamd.id@gmail.com (Ç.M.); 2 Department of Medical Microbiology, Ankara Bilkent City Hospital, 06800 Ankara, Türkiye; irem_tmky@hotmail.com; 3 Department of Medical Microbiology, Health Sciences University, Bilkent City Hospital, 06800 Ankara, Türkiye; bhdogan@yahoo.com; 4 Department of Infectious Diseases and Clinical Microbiology, Medical Faculty, Ankara Yıldırım Beyazıt University, 06800 Ankara, Türkiye

**Keywords:** culture-negative endocarditis, prosthetic valve, non-tuberculous mycobacteria, *Mycobacterium fortuitum*

## Abstract

Background/Objectives: Prosthetic valve endocarditis caused by non-tuberculous mycobacteria is a rare but serious condition and is often associated with delayed diagnosis due to initially negative routine blood cultures with late positivity after prolonged incubation. *Mycobacterium fortuitum*, a rapidly growing mycobacterium, is an uncommon cause of endocarditis but may result in significant morbidity if not promptly identified. Methods: We report a 67-year-old man with prior cardiac surgery who presented 18 months later with recurrent fever, weight loss, and renal dysfunction. Initial blood cultures, echocardiography, and standard imaging were non-diagnostic. Ongoing clinical suspicion prompted extended mycobacterial cultures with prolonged incubation and molecular identification performed at a reference laboratory, which revealed *M. fortuitum*. Results: Antimicrobial susceptibility testing demonstrated susceptibility to amikacin, ciprofloxacin, and clarithromycin, and treatment was initiated with an amikacin-based combination regimen. The patient showed marked clinical and laboratory improvement, including resolution of fever and stabilization of renal function. Conclusions: This case highlights the diagnostic and therapeutic challenges of *M. fortuitum* prosthetic valve endocarditis and underscores the limitations of routine diagnostic methods in culture-negative endocarditis. It also emphasizes the importance of prolonged incubation and targeted microbiological workflows in suspected cases.

## 1. Introduction

Infective endocarditis (IE) is a life-threatening infection characterized by microbial involvement of the endocardial surface of the heart and continues to be associated with high morbidity and mortality despite significant advances in diagnostic imaging, antimicrobial therapy, and surgical management [1,2]. Improvements in echocardiographic techniques, the availability of advanced imaging modalities, and the development of broad-spectrum antimicrobial agents have enhanced diagnostic accuracy and therapeutic outcomes; however, IE remains a complex disease with substantial clinical burden. The disease course is often complicated by systemic embolization, progressive valvular dysfunction, heart failure, and persistent infection, all of which contribute to poor clinical outcomes and frequently necessitate complex multidisciplinary care involving cardiology, infectious diseases, cardiac surgery, and microbiology specialists [3,4,5,6].

Prosthetic valve endocarditis (PVE) represents a particularly severe and challenging form of IE, largely due to the presence of foreign material, altered host immune responses, and the propensity of microorganisms to adhere to prosthetic surfaces and form biofilms. Biofilm formation on prosthetic material significantly reduces antimicrobial penetration and protects pathogens from host immune mechanisms, thereby facilitating persistent infection and increasing the risk of treatment failure [7,8,9]. Consequently, PVE is associated with higher rates of complications, prolonged hospitalization, and increased mortality compared with native valve endocarditis. PVE poses substantial diagnostic and therapeutic challenges, especially when conventional microbiological techniques fail to identify the causative pathogen [10,11,12,13,14,15].

Culture-negative endocarditis (CNE) accounts for up to 20% of all IE cases and remains a major problem in routine clinical practice. The incidence of blood culture–negative endocarditis ranges from 2% to 69%, depending on geographic region, diagnostic methods, and prior antimicrobial exposure, and remains a major challenge in clinical practice [16,17,18,19].

The absence of pathogen growth in standard blood cultures complicates both diagnosis and management, often leading to delays in targeted antimicrobial therapy. The inability to isolate pathogens is most commonly attributed to prior exposure to antimicrobial agents or infection with fastidious or slow-growing organisms that are not readily detected by standard blood culture systems. Among these organisms, nontuberculous mycobacteria (NTM) represent an important but often underrecognized group of pathogens responsible for culture-negative cases [1,9]. Delayed diagnosis of CNE frequently leads to prolonged empirical antimicrobial therapy, increased rates of complications, and suboptimal clinical outcomes, emphasizing the need for heightened clinical awareness and improved diagnostic strategies.

*Mycolicibacterium fortuitum* (formerly *Mycobacterium fortuitum*) is a rapidly growing nontuberculous mycobacterium widely distributed in environmental reservoirs such as soil and water. Following recent taxonomic reclassification, *Mycobacterium fortuitum* has been renamed *Mycolicibacterium fortuitum*. In this manuscript, we refer to the organism as *M. fortuitum* in a clinical context, while acknowledging the updated nomenclature for microbiological precision. It is capable of surviving in a wide range of environmental conditions and is commonly isolated from natural and man-made water sources. Although *M. fortuitum* is well established as a cause of skin and soft tissue infections, postoperative wound infections, and device-related infections, cardiac valve involvement remains exceedingly rare [16]. The organism’s intrinsic resistance to standard disinfectants and its ability to persist in healthcare environments contribute to its association with healthcare-related infections, particularly in postoperative and device-associated settings.

When endocarditis due to *M. fortuitum* occurs, it is most often linked to surgical contamination or colonization of prosthetic material, where biofilm formation plays a critical role in the persistence of infection and resistance to antimicrobial therapy [4,5,6,9,16]. Biofilm-associated infections caused by rapidly growing mycobacteria are notoriously difficult to eradicate and often require prolonged combination antimicrobial therapy guided by susceptibility testing. These characteristics further complicate clinical management and may contribute to delayed microbiological diagnosis and prolonged disease course.

The diagnosis of *M. fortuitum* endocarditis is particularly challenging because clinical manifestations are often nonspecific and may mimic those of more common bacterial causes of IE. Patients frequently present with prolonged fever, constitutional symptoms, and laboratory markers of inflammation, while routine blood cultures frequently remain negative. As a result, diagnosis is commonly delayed, and patients may undergo extensive diagnostic evaluations before the causative organism is identified. Advanced diagnostic approaches, including prolonged incubation of cultures, specialized mycobacterial culture techniques, and molecular identification methods, are often required for definitive diagnosis in such cases.

The growing recognition of atypical pathogens as causes of PVE underscores the importance of maintaining a high index of suspicion in patients presenting with unexplained fever and persistently negative blood cultures following cardiac surgery. Early consideration of NTM and other uncommon organisms may prompt timely use of advanced diagnostic modalities and facilitate earlier initiation of targeted antimicrobial therapy. Despite increasing recognition of nontuberculous mycobacteria as potential causes of prosthetic valve endocarditis, detailed clinical descriptions that integrate diagnostic timing, microbiological workflow, and therapeutic decision-making remain limited in the literature. In particular, reports providing comprehensive documentation of delayed culture positivity, stepwise microbiological identification, and outcomes following conservative medical management without surgery are scarce. Therefore, presentation of such cases may contribute valuable clinical insight into diagnostic strategy, interpretation of microbiological findings, and individualized treatment approaches in rare etiologies of prosthetic valve endocarditis.

While *M. fortuitum* PVE has been previously described, this case is distinguished by the documented initially routine-culture-negative status with delayed blood culture positivity, comprehensive microbiological workflow including molecular confirmation, and a fully documented conservative medical management with 6-month follow-up, providing insights into diagnostic timing and therapeutic decision-making that are not extensively detailed in prior reports. In this report, we present a rare case of late-onset prosthetic valve endocarditis caused by *Mycobacterium fortuitum*. This case highlights the diagnostic challenges associated with initially routine-culture-negative endocarditis and emphasizes the critical role of advanced diagnostic modalities in identifying elusive pathogens. Increased awareness among clinicians, combined with timely use of specialized microbiological and molecular diagnostic techniques, is essential to improve diagnostic accuracy and guide appropriate management strategies in patients with suspected culture-negative prosthetic valve endocarditis.

## 2. Detailed Case Description

A 67-year-old man with a significant cardiovascular history, including coronary artery bypass grafting and mechanical aortic valve replacement performed 18 months earlier, presented with a prolonged clinical course characterized by recurrent systemic symptoms. Over the preceding 12 months, the patient experienced intermittent episodes of high-grade fever, with temperatures reaching up to 40 °C, accompanied by progressive unintentional weight loss and gradual deterioration of renal function. His medical history was further complicated by a recent ischemic stroke and underlying chronic kidney disease, both of which contributed to increased clinical complexity and heightened suspicion for a systemic infectious or inflammatory process.

During multiple hospital admissions, repeated blood cultures obtained during febrile episodes demonstrated transient Gram-variable signals; however, definitive microbial identification could not be achieved using conventional microbiological techniques. Transthoracic and transesophageal echocardiography revealed filamentous, mobile structures adjacent to the mechanical aortic valve prosthesis, raising concern for possible prosthetic valve endocarditis. To further evaluate the extent of suspected infection, positron emission tomography–computed tomography (PET-CT) (GE Healthcare, Milwaukee, WI, USA) was performed and demonstrated increased metabolic activity surrounding the ascending aorta and prosthetic valve, with a reported SUVmax ranging from 6.6 to 8.6. These imaging findings were highly suggestive of an active infectious process involving the prosthetic valve and adjacent vascular structures.

**Figure 1:**
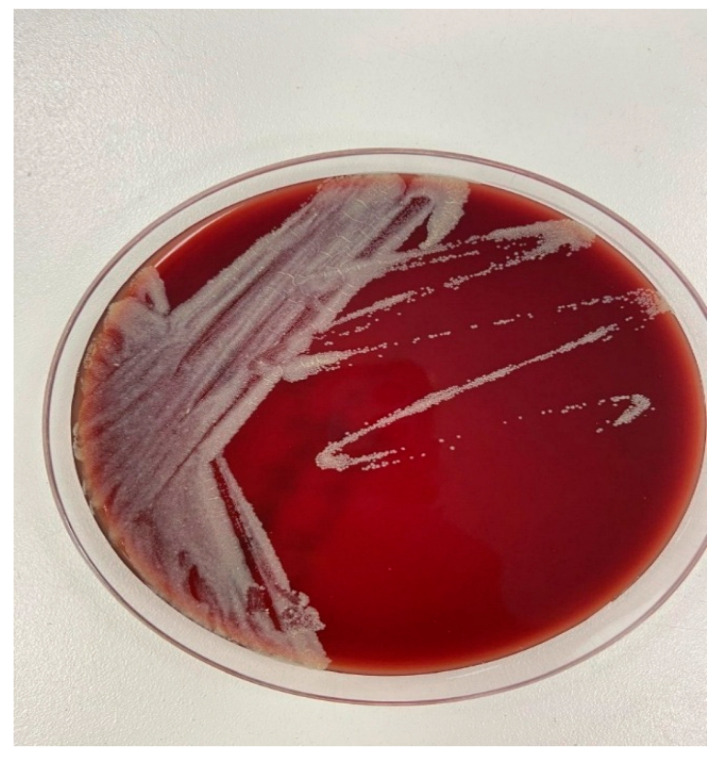
Colony morphology of *Mycobacterium fortuitum* on blood agar after incubation at 37 °C for three days, showing pale, opaque colonies that developed a yellowish coloration upon prolonged incubation.

Blood samples were submitted to the microbiology laboratory for repeated blood culture testing. In the initial sets, bottles gave a positive signal in the automated BacT/Alert 3D system (bioMérieux, Durham, NC, USA); however, no bacterial growth was obtained on subculture. Blood cultures were collected in both aerobic and anaerobic bottles and processed using a continuous automated monitoring system. In subsequent blood culture sets obtained at later time points, bottles again yielded a positive signal, and this time, growth was observed. Bottles yielding a positive signal at the 88th hour were subcultured onto 5% sheep blood agar and chocolate agar plates and incubated at 35–37 °C. After three days of incubation, pale and opaque colonies grew on blood agar, which developed a yellowish coloration upon prolonged incubation (Figure 1). Gram staining of the positive bottle revealed irregularly staining Gram-positive bacilli. Species identification was performed using the VITEK^®^ MS system (bioMérieux, Craponne, France) in at least two independent sets of blood cultures obtained at different time points, and the isolate was confirmed using 16S rRNA gene-targeted PCR at the Public Health Reference Laboratory (Ankara, Türkiye). Ziehl–Neelsen staining was subsequently performed following suspicion of atypical organisms. Ziehl–Neelsen staining and mycobacteria growth indicator tube (MGIT)/Löwenstein–Jensen cultures were also performed. Further molecular identification was conducted using the GenoType Mycobacterium Common Mycobacteria/Additional Species (CM/AS) assay (Hain Lifescience GmbH, Nehren, Germany) based on the reverse hybridization method, which identified the isolate as *Mycolicibacterium fortuitum*. Antimicrobial susceptibility testing was performed using the broth microdilution method for minimal inhibitory concentration (MIC) determination, in accordance with the Clinical and Laboratory Standards Institute (CLSI M24) guidelines (Table 1).

Empirical therapy with meropenem and daptomycin was initiated at presentation because suspected prosthetic valve endocarditis with initially routine-culture-negative findings required guideline-consistent broad-spectrum coverage against staphylococci, enterococci, and Gram-negative pathogens until organism identification. Following microbiological confirmation of *Mycobacterium fortuitum*, treatment was revised to a multidrug regimen targeting rapidly growing mycobacteria. Potential healthcare-associated sources were evaluated through review of surgical records, perioperative sterilization procedures, and hospital water-system surveillance data, none of which identified a source; the infection was therefore presumed related to prior healthcare exposure without a confirmed origin. Although empirical daptomycin plus meropenem was administered during the diagnostic phase, no meaningful clinical or laboratory improvement was observed. In the context of persistent symptoms and the limited availability of evidence-based treatment recommendations for nontuberculous mycobacterial endocarditis, an empirical multidrug regimen was subsequently initiated. This regimen consisted of doxycycline (100 mg orally twice daily), amikacin (7.5 mg/kg intravenously every 24 h), and ciprofloxacin (400 mg intravenously twice daily) while awaiting definitive antimicrobial susceptibility results. The selection of this combination was guided by previously reported in vitro activity against rapidly growing mycobacteria and consideration of the patient’s clinical status and comorbidities.

Antimicrobial susceptibility testing demonstrated sensitivity to amikacin, ciprofloxacin, clarithromycin, linezolid, and moxifloxacin (Table 1), thereby confirming the appropriateness of the selected agents. Therapeutic drug monitoring of amikacin was not available at our institution. However, renal function was closely monitored throughout therapy, with daily assessment of serum creatinine and estimated glomerular filtration rate (eGFR). Amikacin dosing was carefully adjusted according to renal function, and no treatment-related nephrotoxicity was observed. Following receipt of these results, antimicrobial therapy was streamlined to a combination of ciprofloxacin and amikacin. Although susceptibility testing also demonstrated in vitro susceptibility to clarithromycin and linezolid, these agents were not selected because of concerns regarding inducible macrolide resistance in rapidly growing mycobacteria and the preference for a bactericidal regimen in prosthetic valve infection. Under this targeted treatment strategy, the patient exhibited clinical improvement characterized by defervescence within 30 days, CRP decline from 29.7 to 2.1 mg/L, and normalization of leukocyte count, including defervescence and normalization of inflammatory markers, within two weeks of therapy initiation (Table 2).

Based on clinical, microbiological, and imaging findings, the diagnosis fulfilled the modified Duke criteria and contemporary ESC 2023 diagnostic criteria, including one major criterion (positive blood cultures from ≥2 separate sets yielding the same organism) and four minor criteria (prosthetic valve, fever > 38 °C, vascular event, and imaging findings consistent with endocardial involvement) and a six-week course of intravenous antimicrobial therapy was completed. Subsequently, the patient was transitioned to oral moxifloxacin to complete the planned treatment duration. Given the patient’s elevated operative risk and favorable response to medical therapy, a conservative, non-surgical management approach was adopted. The total planned duration of antimicrobial therapy was 12 weeks. Follow-up cardiac computed tomography revealed no evidence of residual vegetation. Upon completion of the three-month treatment regimen, the patient was scheduled for close outpatient follow-up to monitor for potential relapse, and at the most recent outpatient evaluation 6 months after completion of therapy, no clinical, laboratory, or imaging evidence of relapse was observed. Given the elevated relapse risk associated with medical-only management of prosthetic valve endocarditis, the patient was followed closely for 6 months after completion of therapy, during which no clinical, laboratory, or imaging evidence of relapse was observed. The total treatment duration of 12 weeks was selected based on available evidence from published case series of nontuberculous mycobacterial endocarditis and expert opinion suggesting prolonged multidrug therapy due to the organism’s biofilm-forming capacity and high relapse potential. Given the absence of standardized treatment duration recommendations for NTM endocarditis, therapy length was individualized according to clinical response, microbiological clearance, inflammatory marker normalization, and radiological improvement. Transition from intravenous to oral therapy was performed after the patient achieved sustained defervescence, clinical stabilization, declining inflammatory markers, and no evidence of persistent bacteremia.

The total duration of antimicrobial therapy was 12 weeks. Intravenous therapy consisted of amikacin plus ciprofloxacin administered for 6 weeks. Following completion of the intravenous phase and after achievement of sustained clinical improvement, including defervescence, normalization of leukocyte count, and marked decline in inflammatory markers, treatment was transitioned to oral moxifloxacin, which was continued for an additional 6 weeks to complete the planned course. Treatment endpoints included sustained clinical stability, absence of bacteremia, normalization of inflammatory parameters, and radiologic improvement on follow-up imaging. During follow-up, the patient remained afebrile and demonstrated progressive clinical recovery. Serial laboratory evaluations showed marked decline in inflammatory markers with normalization of leukocyte count and sustained reduction of C-reactive protein levels. Follow-up echocardiography revealed no detectable vegetations or new valvular abnormalities, confirming objective clinical and radiological response to therapy. After completion of therapy, the patient was followed for 6 months, during which no clinical, laboratory, or imaging evidence of relapse was detected.

## 3. Discussion

Prosthetic valve endocarditis (PVE) remains a diagnostically and therapeutically complex clinical entity, particularly when it develops long after valve implantation. Late-onset PVE is often associated with subtle and nonspecific clinical findings, including low-grade or intermittent fever, constitutional symptoms, and laboratory abnormalities that may be attributed to alternative diagnoses. These nonspecific manifestations frequently contribute to delays in diagnosis and initiation of appropriate therapy, which in turn adversely affect patient outcomes. The spectrum of causative microorganisms varies substantially according to the timing of disease onset, reflecting differences in perioperative exposure, host-related factors, and underlying mechanisms of infection.

Conventionally, a 12-month cutoff has been used to distinguish early from late PVE. In the early postoperative period, defined as within the first two months following valve implantation, *Staphylococcus aureus* predominates as the leading causative pathogen, followed by coagulase-negative staphylococci, Gram-negative bacilli, and *Candida* species. During the intermediate period between 2 and 12 months, *Streptococcus* spp., *S. aureus*, coagulase-negative staphylococci, and *Enterococcus* spp. are more frequently identified [5,10]. Beyond this timeframe, the microbiological profile may shift toward less common and atypical organisms, particularly in patients with ongoing healthcare exposure or implanted prosthetic material, further complicating diagnostic evaluation. While staphylococci and enterococci remain the most frequently reported causative agents of prosthetic valve endocarditis, the recent literature emphasizes the importance of considering fastidious and atypical pathogens, including nontuberculous mycobacteria, particularly in late-onset or culture-negative cases [13,15].

*Mycobacterium fortuitum* is an environmental, rapidly growing nontuberculous mycobacterium (NTM) widely distributed in natural and man-made water sources as well as soil. Although it rarely causes clinically significant disease in immunocompetent individuals, it may lead to severe, persistent, and invasive infections in immunocompromised hosts, postoperative patients, or individuals with implanted medical devices. NTMs implicated in PVE are typically healthcare-associated and have been linked to contaminated cardioplegia solutions, hospital water systems, inadequately sterilized surgical instruments, and prosthetic valve preservation media [3,4,9,12]. These observations underscore the importance of stringent infection control measures and highlight the potential for environmental mycobacteria to cause serious postoperative infections.

In recent years, global outbreaks of *Mycobacterium chimaera* infections associated with heater–cooler devices used during cardiothoracic surgery have further emphasized the ability of environmental mycobacteria to cause serious, delayed, and often life-threatening cardiovascular infections [4]. These outbreaks have increased awareness of NTMs as potential causes of culture-negative endocarditis and have prompted renewed interest in improving surveillance, diagnostic strategies, and preventive measures in cardiac surgery settings [13].

Endocarditis due to *M. fortuitum* is exceedingly rare, with only a limited number of adult cases described in the literature to date. Published reports indicate that the majority of cases involve prosthetic valves, particularly mechanical valves, and are frequently associated with prior cardiac surgery or invasive cardiovascular procedures. A comprehensive, independently verified dataset including cases identified from 2000 through January 2026 is presented in Table 3. The previously published summary derived from the Olalla review is provided separately in Table 4 for comparison. Previously reported cases of *Mycobacterium fortuitum* complex endocarditis demonstrate a clear predominance of male patients, with a mean age in the mid-forties. The summary presented in Table 3 was derived from previously published literature reviews summarizing reported cases of *Mycobacterium fortuitum* complex endocarditis rather than from an independent systematic search performed by the authors. The literature summary presented in Table 3 was compiled from previously published case reports, case series, and review articles identified through targeted searches of major biomedical databases (including PubMed/MEDLINE, Scopus, and Google Scholar) using combinations of keywords such as “*Mycobacterium fortuitum*,” “non-tuberculous mycobacteria,” “infective endocarditis,” and “prosthetic valve endocarditis.” Articles were selected based on relevance to clinical presentation, microbiological diagnosis, treatment, and outcomes. This review was narrative in nature and was not intended as a formal systematic review; therefore, the results should be interpreted with awareness of potential publication and selection bias. To avoid duplication, each case was carefully reviewed and cross-checked using author name, publication year, and patient characteristics. When overlap between review articles and original case reports was suspected, only the original report was included. The updated literature search covered publications from 2000 to January 2026 using PubMed. Cases identified after the Olalla review were added to Table 3. The data summarized in the Olalla review are presented separately in Table 4 and were not combined with our independently collected cases. “*Mycobacterium fortuitum*,” “rapidly growing mycobacteria,” and “endocarditis.” Only reports describing human cases with microbiologically confirmed diagnosis were included. Non-clinical studies, duplicate reports, and publications lacking sufficient clinical data were excluded. Reference lists of relevant articles were also screened to identify additional eligible reports. The majority of infections occurred in patients with prosthetic heart valves, particularly mechanical valves, while native valve involvement was rare. Aortic valve involvement was more frequently reported than mitral or other valve localizations. Blood culture positivity was observed in only a proportion of cases, whereas culture-negative presentations were more common among patients with bioprosthetic valves. Surgical valve replacement was performed in less than half of the reported cases. Reported mortality rates in published case series appear high; however, these estimates are derived from small numbers of reported cases and should be interpreted cautiously due to potential publication bias, heterogeneity in case definitions, and incomplete outcome reporting (Table 3) [1]. Predisposing factors reported in the literature include immunosuppressive conditions, chronic hemodialysis, intravenous drug use, balloon valvotomy, and percutaneous coronary interventions [4,6]. The clinical course is typically subacute, with symptom onset occurring between 1 and 12 months after cardiac surgery, most commonly around the third postoperative month. Patients often present with persistent or recurrent fever, fatigue, weight loss, and signs of congestive heart failure. Embolic complications involving the central nervous system or peripheral vasculature have also been reported and may significantly contribute to morbidity and long-term disability [11].

In patients with mechanical heart valves, *M. fortuitum* represents a particularly uncommon etiology of PVE. One of the most important pathogenic features of this organism is its ability to form highly adherent biofilms on prosthetic material. Biofilm formation significantly reduces antimicrobial penetration and impairs host immune clearance, thereby complicating eradication and predisposing to chronic or relapsing infection [8]. These characteristics further explain the poor response to empirical antimicrobial regimens and the frequent need for prolonged, targeted therapy guided by antimicrobial susceptibility testing.

In the present case, repeated conventional blood cultures were initially negative and only became positive after prolonged incubation and initial echocardiographic findings were inconclusive, highlighting the diagnostic challenges associated with culture-negative endocarditis. Similar findings have been consistently reported in the literature, where nontuberculous mycobacterial endocarditis is frequently associated with negative conventional blood cultures, often necessitating molecular or advanced microbiological diagnostic approaches [13,14]. The absence of microbiological confirmation using routine methods necessitated the use of advanced diagnostic strategies. Definitive pathogen identification was ultimately achieved through the application of advanced microbiological and molecular diagnostic techniques, emphasizing their indispensable role in contemporary clinical practice, particularly in patients with suspected PVE and negative standard cultures.

Treatment of *M. fortuitum* infections remains challenging and generally requires prolonged combination antimicrobial therapy guided by in vitro susceptibility testing. Commonly used regimens include agents such as amikacin, doxycycline, and fluoroquinolones administered over extended durations. Although surgical intervention is frequently considered necessary for definitive management of prosthetic valve infections, favorable outcomes may be achieved with medical therapy alone in carefully selected high-risk patients who demonstrate an adequate clinical and microbiological response to antimicrobial treatment [4]. These observations highlight the importance of individualized, multidisciplinary decision-making in the management of rare causes of PVE.

A recent systematic review evaluating reported cases of nontuberculous mycobacterial infective endocarditis demonstrated very high mortality rates approaching 45%, despite the majority of patients receiving combined surgical and antimicrobial therapy, underscoring the aggressive clinical behavior and therapeutic challenges associated with these infections [20].

Management of NTM endocarditis remains controversial and is associated with substantial mortality despite aggressive treatment strategies. A recent systematic review reported high mortality even among patients receiving combined surgical and antimicrobial therapy, highlighting the aggressive nature of these infections. Current evidence suggests that optimal management often requires prolonged multidrug therapy guided by susceptibility testing, consideration of early surgical intervention when feasible, and, in selected high-risk cases, chronic suppressive antimicrobial therapy. However, standardized treatment algorithms remain lacking, and management decisions should be individualized within a multidisciplinary framework.

Supporting this observation, one of the earliest published cases documented successful non-surgical management of prosthetic valve endocarditis caused by *Mycobacterium fortuitum*, indicating that conservative treatment may represent a reasonable option in selected patients when surgery is contraindicated or high risk [21].

This case reinforces the importance of considering rapidly growing mycobacteria in patients with prosthetic material and unexplained fever, particularly when blood cultures show delayed positivity or atypical staining characteristics.

Although previously reported cases of *M. fortuitum* prosthetic valve endocarditis exist, the distinguishing aspects of this case include detailed documentation of delayed culture positivity, stepwise microbiological identification, and successful individualized medical management in a high-risk surgical patient. A total of 29 cases of *Mycobacterium fortuitum* complex endocarditis, including the present case, have been reported in the literature. Prosthetic valve involvement remains predominant (72.4%). The overall mortality decreased slightly to 58.6% after inclusion of the present case. Detailed individual case characteristics, management strategies, and outcomes are summarized in Table 4. Surgical intervention was associated with lower mortality compared to medical therapy alone. These findings reinforce the aggressive clinical course of *M. fortuitum* endocarditis and the potential benefit of combined medical–surgical management (Table 3).

**Table 4:** Main characteristics of the cases.

Author (Reference)	Age	Sex	Type of Valve	Localization	Blood Cultures	Isolation from Other Sources	Valve Replacement	Antibiotic Therapy	Death
Olalla [1]	57	F	Metallic	Mitral	-	Sternum	Yes	Imipenem + amikacin	Yes
Gnanenthiran [3]	65	F	Bioprosthetic	Mitral	+	-	No	Amikacin + ciprofloxacin/moxifloxacin	No
Spell [6]	47	M	Native	Aortic	+	-	No	Amikacin + ciprofloxacin + cefoxitin	Yes
Bosio [7]	72	M	Metallic	Aortic	+	Porcine aortic root tissue	No	Amikacin + trimethoprim/sulfamethoxazole + moxifloxacin	No
Kunin [21]	75	M	Biological	Aortic	+	-	Yes	Meropenem + amikacin	No
Narasimham [22]	57	M	Metallic	Aortic	+	Sternum	No	Ethionamide + isoniazid + rifampin	Yes
Galil [23]	66	M	Native	Tricuspid	+	Lung	No	Clarithromycin + ciprofloxacin	No
Creagh [24]	74	M	Biological	Mitral	+	Vertebra	No	Sulfadiazine + ampicillin + chloramphenicol	Yes
Altmann [25]	45	M	Metallic	Aortic	+	–	No	-	Yes
Singh [26]	54	F	Native	Aortic	+	–	No	Trimethoprim sulfamethoxazole/ciprofloxacin/clofazimine + amikacin	Yes
Noremberg [27]	46	M	Metallic	Aortic	-	-	Yes	Sulfadiazine + İsonazid + Streptomycin + ethambutol	Yes
Kuritsky [28]	43	M	Biological	Mitral	-	-	No	-	Yes
Rumisek [29]	25	F	Biological	Mitral	-	-	Yes	Isoniazid + erythromycin	Yes
Levy [30]	55	M	Biological	Aortic	-	-	No	-	No
Viscidi [31]	55	M	Metallic	Mitral	+	-	No	Isoniazid + rifampin + erythromycin + ethambutol/erythromycin + amikacinþ	Yes
Chow [32]	45	F	Metallic	Aortic	+	Sternum	Yes	Amikacin + ciprofloxacin	Yes
Repath [33]	43	M	Metallic	Aortic and mitral	+	Urinary catheter	No	Penicillin + gentamicin/kanamycin + erythromycin	Yes
Vail [34]	21	F	Metallic	Aortic	+	-	Yes	Amikacin + ciprofloxacin + imipenem clarithromycin	No
Alvarez-Elcoro [35]	27	F	Metallic	Mitral	-	Bone marrow	Yes	Amikacin + rifampin	Yes
DoCarmo [36]	40	M	Biological	Aortic	-	-	Yes	Isoniazid + rifampin + pyrazinamide	Yes
Kuruvila [37]	20	F	Native	Mitral	+	CSF	No	Amikacin + azithromycin + rifampin	Yes
Siu [38]	78	F	Native	Pacemaker	-	Pectoral Region	No	Vancomycin + amikacin + levofloxacin	No
Bush [39]	47	F	Metallic	Aortic	-	-	No	Amikacin + clarithromycin + tigecycline	Yes
Natsag [40]	49	F	Native	Aortic and tricuspid	+	-	No	linezolid + ciprofloxacin + Trimethoprim sulfamethoxazole	No
Mulhall [41]	64	F	Bioprosthetic	Pulmonic	+	Tracheal aspirate	No	Amikacin + imipenem,	Yes
Al Zoubi [42]	26	F	Native	Tricuspid	-	Implantable cardioverter-defibrillator	No	Azithromycin + Levofloxacin + imipenem then TMX/SMX + doxycyclin	No
Vaidya [43]	66	F	Native	Mitral	+	-	No	Imipenem-cilastatin + amikacin + Levofloxacin	No
Hurley [44]	70	F	Bioprosthetic	Mitral	+	-	No	Amikacin + imipenem-cilastin, + levofloxacin then doxycycline	No
Our present case	67	M	Metallic	Aortic	+	-	No	Doxycycline + amikacin + ciprofloxacin then moxifloxacin	No

M, male; F, female; CSF, cerebrospinal fluid.

## 4. Conclusions

This case underscores the necessity of recognizing *Mycobacterium fortuitum* as an infrequent yet clinically meaningful etiological agent of prosthetic valve endocarditis. In patients who develop persistent or otherwise unexplained febrile syndromes accompanied by repeatedly negative blood culture results following cardiac procedures, a heightened and sustained level of clinical suspicion for nontuberculous mycobacteria and other atypical or fastidious microorganisms is warranted.

In cases of culture-negative endocarditis, the early and systematic integration of advanced molecular and sequencing-based diagnostic modalities is essential to ensure accurate pathogen identification. Timely and precise diagnosis facilitates the prompt initiation of appropriately targeted antimicrobial therapy and contributes to improved clinical response and overall patient outcomes.

## Figures and Tables

**Table 1:** Antibiotic Susceptibility of *M. fortuitum* Isolate with CLSI M24 criteria for sensitivity.

Antibiotic	MIC (µg/mL)	Interpretation
Amikacin	2	S
Cefoxitin	64	I
Ciprofloxacin	0.25	S
Clarithromycin	0.5	S
Doxycycline	16	R
Imipenem	8	I
Linezolid	4	S
Moxifloxacin	0.5	S
Trimethoprim-sulfamethoxazole	4	R

Interpretation key for the antibiogram: S = Susceptible (antibiotic is effective), I = Intermediate (limited effectiveness), R = Resistant (antibiotic is not effective).

**Table 2:** Trend of Acute Phase Reactants During Treatment.

Treatment Day	C-Reactive Protein (CRP) mg/L	Procalcitonin (PCT) µg/L	White Blood Cell (WBC) 10^9^/L, % Neutrophils (NEU%)
DAY 1	29.7	0.58	7.18, 72%
DAY 3	35.9	0.91	6.03, 79%
DAY 7	42.1	0.92	3.39, 68%
DAY 14	3.1	0.17	5.41, 57%
DAY 30	2.1	0.14	6.12, 54.8%

CRP: C-Reactive Protein, PCT: Procalcitonin, WBC: White Blood Cell, NEU: Neutrophils.

**Table 3:** Summary of reported cases of *Mycobacterium fortuitum* complex endocarditis (n = 29) (data adapted from previously published literature).

Characteristic	Value
Total cases	31
Male	16 (52%)
Female	15 (48%)
Mean age (range)	52 years (20–78)
Prosthetic valves	23 (74%)
Mechanical	11
Bioprosthetic	12
Native valves	7 (23%)
Device-related	1 (3%)
Aortic involvement	14 (45%)
Mitral involvement	10 (32%)
Tricuspid involvement	3 (10%)
Pulmonic involvement	1 (3%)
Multiple valve involvement	2 (6%)
Blood culture positive	15 (48%)
Surgery performed	11 (35%)
Overall mortality	17 (55%)

## Data Availability

The data presented in this study are available upon request from the corresponding author due to patient privacy considerations.

## Abbreviations

CM/AS	Common mycobacteria/additional species
CNE	Culture-negative endocarditis
CRP	C-Reactive Protein
CLSI-M24	Clinical and Laboratory Standards Institute document M24, Guidelines for Susceptibility Testing of Mycobacteria, Nocardiae, and Other Aerobic Actinomycetes
IE	Infective endocarditis
MALDI-TOF	Matrix-Assisted Laser Desorption/Ionization–Time of Flight
MGIT	Mycobacteria Growth Indicator Tube
mNGS	Metagenomic Next-Generation Sequencing
NEU%	Neutrophil Percentage
NTM	Nontuberculous Mycobacterium
PCR	Polymerase Chain Reaction
PET-CT	Positron Emission Tomography–Computed Tomography
PCT	Procalcitonin
PVE	Prosthetic Valve Endocarditis
rRNA	Ribosomal RNA
SUVmax	Maximum Standardized Uptake Value
WBC	White Blood Cell Count
